# The influence of drying and storage conditions on the volatilome and cannabinoid content of *Cannabis sativa* L. inflorescences

**DOI:** 10.1007/s00216-024-05321-w

**Published:** 2024-05-03

**Authors:** Natasha Damiana Spadafora, Simona Felletti, Tatiana Chenet, Tiziana Maria Sirangelo, Mirco Cescon, Martina Catani, Chiara De Luca, Claudia Stevanin, Alberto Cavazzini, Luisa Pasti

**Affiliations:** 1 Department of Chemical, Pharmaceutical, and Agricultural Sciences, University of Ferrara, Via Luigi Borsari 46, 44121 Ferrara, Italy; 2 Department of Environmental and Prevention Sciences, University of Ferrara, Via L. Borsari 46, 44121 Ferrara, Italy; 3 ENEA-Italian National Agency for New Technologies, Energy and Sustainable Economic Development-Division Biotechnologies and Agroindustry, 00123 Rome, Italy; 4 Council for Agricultural Research and Economics, CREA, Via Della Navicella 2/4, 00184 Rome, Italy

**Keywords:** Volatile organic compounds, Terpenes, SPME, GC × GC, Cannabinoids, HPLC, PCA, Hierarchical cluster analysis

## Abstract

The increasing interest in hemp and cannabis poses new questions about the influence of drying and storage conditions on the overall aroma and cannabinoids profile of these products. Cannabis inflorescences are subjected to drying shortly after harvest and then to storage in different containers. These steps may cause a process of rapid deterioration with consequent changes in precious secondary metabolite content, negatively impacting on the product quality and potency. In this context, in this work, the investigation of the effects of freeze vs tray drying and three storage conditions on the preservation of cannabis compounds has been performed. A multi-trait approach, combining both solid-phase microextraction (SPME) two-dimensional gas chromatography coupled to mass spectrometry (SPME-GC × GC–MS) and high-performance liquid chromatography (HPLC), is presented for the first time. This approach has permitted to obtain the detailed characterisation of the whole cannabis matrix in terms of volatile compounds and cannabinoids. Moreover, multivariate statistical analyses were performed on the obtained data, helping to show that freeze drying conditions is useful to preserve cannabinoid content, preventing decarboxylation of acid cannabinoids, but leads to a loss of volatile compounds which are responsible for the cannabis aroma. Furthermore, among storage conditions, storage in glass bottle seems more beneficial for the retention of the initial VOC profile compared to open to air dry tray and closed high-density polyethylene box. However, the glass bottle storage condition causes formation of neutral cannabinoids at the expenses of the highly priced acid forms. This work will contribute to help define optimal storage conditions useful to produce highly valuable and high-quality products.

**Graphical Abstract:**

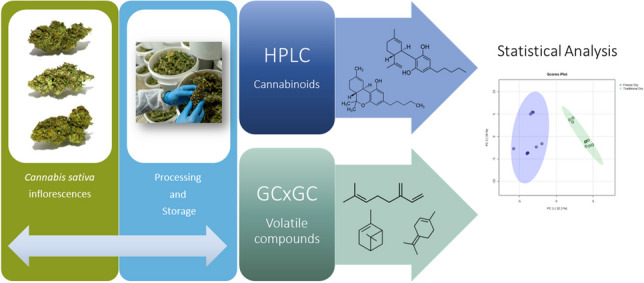

**Supplementary Information:**

The online version contains supplementary material available at 10.1007/s00216-024-05321-w.

## Introduction

Cannabis (*Cannabis sativa* L.) is a plant of the Cannabaceae family originating from Central Asia and widely distributed around the world, due to its climatic and territorial adaptability [1]. It is one of the oldest cultivated multipurpose crops; in fact, it can be classified as fibre crop (hemp), producing cellulosic and woody fibres, and as drug crop (medicinal cannabis) which is used for therapeutic purposes [2].

Over 500 secondary metabolites have been reported in cannabis plants, mainly present in inflorescences and leaves, including cannabinoids and non-cannabinoids like phenols and flavonoids, terpenes, and alkaloids [3, 4]. The content, composition, and kinetics of secondary metabolites is highly dependent on the cannabis genotype, environment, and pre- and post-harvest conditions [5–7]. The most abundant cannabinoids found in cannabis are ∆^9^-tetrahydrocannabinolic acid (∆^9^-THCA), cannabidiolic acid (CBDA), cannabichromenic acid (CBCA), and their precursor cannabigerolic acid (CBGA). However, the exposure to high temperatures, light and air leads to a decarboxylation reaction, producing neutral active form [8]. Cannabidiol (CBD) and ∆^9^-tetrahydrocannabinol (∆^9^-THC) are the two neutral cannabinoids with the highest reported bioactivity [9]. CBD is documented to have antipsychotic, antidepressant, anxiolytic, and anti-inflammatory effects and to act against various types of cancer [10–14]. THC is the main psychoactive compound found in cannabis, and for this reason, its content is strictly regulated by the law. Nevertheless, it has been proved to have anti-inflammatory efficacy as well as having effect on Alzheimer [15] Parkinson [16], and diabetes diseases [17]. Furthermore, cannabigerol (CBG) and cannabichromene (CBC) are reported to have anti-inflammatory therapeutic effects [18].

The generally accepted classification of cannabis is based on the content of CBD and THC: drug-type chemotypes are characterised by higher level of THC compared to CBD, whereas the opposite is true for fibre-type chemotypes [19, 20]. Recently, the Center for Genetic Resources of Cannabis (CGRC, www.medcanabase.org) published a detailed classification [21]: THC-chemotype I (high THC/CBD, with a THC content > 0.3–20% and CBD < 0.5%); an intermediate-chemotype II (THC and CBD ratio is 0.5–2); CBD-chemotype III (high CBD/THC, with mainly CBD, and THC < 0.3% or undetectable). Two other chemotypes have been tentatively added to this classification, namely, chemotype IV and V. The former is characterised by high CBG content and contains CBD in traces, while chemotype V is characterised by undetectable amounts of any cannabinoids. Medical varieties include both THC and CBD chemotypes [21].

Cannabis is also characterised by a rich volatile organic compounds (VOCs) profile: about 200 compounds have been reported to contribute to the complex scent of the genus [22]. Among them, one can find terpenoids, aldehydes, and esters. Terpenoids are abundant secondary metabolites, with mono and sesquiterpenes, as well as isoprenes, being predominant [23]. Terpenes are responsible for the typical cannabis aroma and are involved in the plant defence system against pathogens [24, 25]. They have shown important anxiolytic and antidepressant effects [26] and are highly priced for their pharmacological properties [4]. To cite few, β-myrcene is a common flavouring and aroma agent used in the manufacture of food and beverages; its anxiolytic, antioxidant, anti-inflammatory, analgesic, and properties are also considered useful in the pharmaceutical industry [27]. Furthermore, α-pinene plays a crucial role in the fragrance and flavour industry and has been used in recent research to synthesise new chemical entities with pharmacological and herbicidal activities [28]. Moreover, d-limonene is prevalently used as a perfuming and a flavouring agent [29]; however, it has shown immune-modulatory properties, antitumor, and anti-inflammatory effects [30]. Terpinolene is widely used as a flavouring agent in the industry [31]. Moreover, it is also well known for its biological effects, including antioxidant, larvicide, and insecticide properties [31]. Furthermore, camphene has been widely used as a flavour additive and a fragrance ingredient in cosmetics and food industries, and in one recent investigation, its biological properties, including antibacterial, antifungal, anticancer, antioxidant, antiparasitic, antidiabetic, and anti-inflammatory activities, were confirmed [32].

Several studies have shown that cannabinoid therapeutic activity can be enhanced either by the presence of other cannabinoids (“intra-entourage effect”) or of terpenes and terpenoids (“inter-entourage effect”), leading to synergistic interactions [33–35]. Cannabinoid-terpenoid synergy could be advantageous for the development of novel medications and therapeutic products for the treatment of several disorders, including inflammation, depression, and cancer [36]. In this context, the preservation of the initial content of both VOCs and cannabinoids present in freshly harvested cannabis inflorescences becomes of pivotal importance. As a matter of fact, the exposure to light, high temperatures, and air as well as favouring the decarboxylation of acidic cannabinoids to neutral ones [8] induces the degradation of sesquiterpenes and bigger terpenes into monoterpenes. Moreover, monoterpenes undergo degradation more easily than larger terpenes, due to their higher volatility [37]. This poses new concerns about the correct treatment, preservation, and storage conditions of cannabis inflorescences.

Indeed, fresh harvested cannabis is characterised by a rapid deterioration with consequent loss of precious secondary metabolites due to the high moisture content (78–80%). The most common preservation technique to retain bioactive compounds involves reduction of water content in inflorescences and leaves, achieved by drying. [38]. The choice of the drying method greatly influences the final content of volatile compounds, such as terpenes, and cannabinoids [38–40]. During the drying process, phytochemicals may either decrease or increase, depending on the plant species, and the formation of new chemical compounds may occur [41].

A common drying method is “screen/tray drying” which consists in the removal of long leaves around the flowering area, resulting in trimmed flowers of 10–15 cm in length, and the positioning of the manicured flowers onto trays/screens. Temperature and humidity conditions of the environment are strictly controlled between 18–21 °C and 50–55%, respectively, and the entire process lasts about 5 days [42, 43]. However, drying processes are time-consuming and require a dedicated inspection, as well as proper maintenance. Cutting edge technologies used for other crops, such as vacuum freeze drying, may find employment for cannabis drying process [44]. In vacuum freeze drying, samples are placed in a cold chamber and frozen by reducing the temperature below − 40 °C, so that water freezes into small ice crystals. Once crystals have formed, the pressure is reduced to create vacuum. Drying is complete within 24 to 48 h. This method may inhibit microbial activities, preserve the sample structure by inhibiting enzymatic processes, and affect trichome structure [45, 46]. Nevertheless, the preservation of important traits such as VOCs is still not fully proven.

Quality and potency of cannabis may be also affected by post-harvest storage conditions. Storage temperature, time, light exposure, and packaging material are the main factors affecting the chemical composition of cannabis by disrupting the biosynthesis of secondary metabolites [40, 47, 48]. Storage studies performed under controlled conditions, in air-tight bags in darkness showed preservation of secondary metabolites [48]. However, it has been recently observed that cannabinoids show different degradation kinetics and thermal stability depending on their molecular structure [49, 50]. This indicates that each cannabis chemovar may exhibit unique degradation paths based on the content and type of cannabinoids. Nevertheless, no comprehensive information on the comparative analysis of VOC profiles and cannabinoid content on cannabis dried and stored under different conditions is present. The aim of this work was to compare and to evaluate the effectiveness of novel and conventional drying technologies and the effects of different storage conditions on cannabis inflorescences. In detail, inflorescence samples of *C. sativa* were subjected to the conventional “tray drying” method and compared to the novel “vacuum freeze drying” processing. The influence of storage method was then evaluated under controlled temperature and humidity conditions in darkness upon three conditions, namely, open to air dry tray, closed high-density polyethylene (HDPE) box, and closed brown glass bottle. To account for the variability in the composition, content, and degradation of samples, the data from three *C. sativa* commercial varieties (Kompolti, Eletta Campana, and Silvana) were pooled.

Inflorescence quality upon drying and storage condition was evaluated for the first time by the analysis of VOC profiles and cannabinoid content through a multi-trait approach which combines both gas and liquid chromatography. VOCs were analysed using an enhancement of solid-phase microextraction (SPME) gas chromatography mass spectrometry (GC–MS), namely SPME-GC × GC–MS. In this two-dimensional configuration, the chromatographic resolution is improved by coupling two GC columns of different selectivity. Therefore, VOCs with close retention time in the first column can be separated in the second column based on different chemical properties, while the detailed analysis of cannabinoids was achieved by high-performance liquid chromatography (HPLC) operated under reversed-phase conditions using a C18 column. This technique is indeed the most used and effective for the separation and resolution of both acid and neutral cannabinoids contained in cannabis sample and extract [51].

This study will contribute to help define optimal storage conditions useful to produce highly valuable and high-quality products.

## Materials and methods

### Plant material

Inflorescences of three *C. sativa* varieties were kindly provided by Società Agricola Jure S.r.l., San Giovanni in Fiore (CS), Calabria, Italy. Two varieties belong to the CBD-chemotype III, Kompolti (K) and Silvana (S), while the third one, Eletta Campana (E), is included in the CBG-chemotype IV. Plants were grown in the same field under the same environmental and agricultural practices. Sampling was carried out in the 2022 autumn season. Mature inflorescences were collected manually and for each variety 20 kg of inflorescences were trimmed from fan leaves, mixed, and subjected to drying.

### Drying conditions

For each cannabis variety, one batch of inflorescences was immediately transferred to the “tray drying” (TD) room maintained at 18 °C and 55% humidity for 6 days, typical time required for the moisture loss to reach a plateau. A second batch of inflorescences was frozen in liquid nitrogen and stored at − 80 °C overnight prior “freeze drying” (FD). Frozen samples were placed on a freeze dryer (Alpha 1–2 LSC, Christ, Germany) set at − 60 °C and 0.01 mbar for 24 h.

### Storage conditions

Dried cannabis inflorescences of each variety were stored in a dark chamber with controlled temperature and humidity (18 °C and 60%, respectively) for 6 months. The storage conditions, selected in agreement with market necessities as suggested by the producer, were (i) 500 mL V airtight box made of high-density polyethylene (HDPE), (ii) open to air dry tray (AT), and (iii) 250 mL lid tight brown glass bottle (GB). The amount of sample used for the storage conditions ranged from ~ 50 g in GB to100 g for HDPE and AT.

### HS-SPME procedure

VOC collection and extraction was essentially as described in Cicaloni et al. (2022) [5] with minor modifications. Quantities of 0.5 g of each cannabis variety were weighted in 20 mL screw top glass vial with PTFE-coated silicone septum.

A SPME fibre (Supelco, Bellefonte, PA, USA) of 1 cm in length, coated with 50/30 µm divinylbenzene/carboxen/polydimethylsiloxane (DVB/CAR/PDMS) was used for the VOCs extraction. Before use, the fibre was conditioned as suggested by the manufacturer. SPME fibre blanks were performed at the start and at set points of each sequence to make sure that carry-over was absent. The entire extraction process was carried out using a robotic sampling platform (EST Analytical, USA). The sample was equilibrated for 10 min at 50 °C under stirring at 800 rpm. The extraction was performed by exposing the fibre to the headspace of the sample for 50 min at 50 °C to allow a wider range of VOCs to be extracted. After this time, the fibre was thermally desorbed in the GC injector at 250 °C for 5 min with a split ratio of 1:20. An alkane mixture (C7-C30, MilliporeSigma, USA) was used for system quality control and linear retention index calculation.

### GC × GC–MS analysis of VOCs

The analysis of the VOCs was performed using a reverse-inject differential flow modulator (Agilent Technologies, USA) mounted on a 7890B GC system (Agilent Technologies). Total modulation period was set to 3 s with a flash time of 100 ms. The column set consisted of the primary column HP-5 ms Ultra Inert column (20 m × 0.18 mm × 0.18 µm, Agilent) and the secondary column DB-17 ms (2.5 m × 0.25 mm × 0.25 µm, Agilent). The secondary column flow was split through a passive splitter plate (Agilent) to two uncoated capillary columns, one connected to the mass spectrometer (0.52 m × 0.1 mm), and the other one connected to a FID (0.9 m × 0.18 mm) for a total split ratio of 1:4 ideal for the MS detector. The GC oven was programmed at 40 °C (hold 1 min) and increased to 230 °C (hold 2 min) at 4 °C/min. Helium was used as carrier gas, with a flow of 0.5 mL/min through the first column and 10 mL/min through the second column. Detection was performed using a 5977B mass spectrometer (Agilent Technologies) operated at an ion source temperature of 250 °C and a transfer line to 280 °C. Ions were collected in a mass range of m/z of 45–350 and an acquisition frequency of 50 Hz.

### Elaboration of GC × GC–MS data

GC × GC–MS data were processed using ChromSpace + software v. 2.1.4 (SepSolve Analytical Ltd, UK). Integrated peaks were screened against the NIST20 spectral library. Putative identification of compounds was carried out on matches of their spectra above 80% and LRI within a ± 20 range compared to the one reported in the NIST library. Peak areas of the compounds were normalised using probabilistic quotient normalisation (PQN) [52], and logarithmic transformation was applied to reduce weight of large components.

### HPLC analysis of cannabinoids

The samples were prepared as follows: 0.05 g of grounded cannabis inflorescence was weighted in 15 mL glass vials. After addition of 5 mL of pure HPLC grade methanol, the samples were ultrasonicated in a Elmasonic S 100 H bath (Elma Schmidbauer GmbH, DE) for 15 min and centrifuged at 6000 rpm for 10 min. The supernatant was filtered with a PVDF membrane filter, 13 mm diameter, 0.2 µm pore size mounted on a 6-mL plastic syringe.

All measurements were carried out in triplicates on an Agilent UHPLC Infinity 1290 equipped with a binary pump, autosampler, column compartment, and a diode array detector (DAD) under reversed-phase conditions.

A 150 × 2.1 mm Raptor ARC-18 column packed with 2.7 µm particles was kindly supplied by Restek (Cernusco sul Naviglio, Milan). Mobile phases were orthophosphoric acid (pH = 2.2) and pure acetonitrile. The gradient programme was as follows: 0–4 min at 68% ACN, 4–10 min from 68 to 90% ACN, 10–11 min at 90% ACN, 11.1–13 min 68% ACN. The flow rate was 0.4 mL/min. Injection volume was 1 μL. The temperature was set at 25 °C, and detection wavelengths were 228 and 306 nm for neutral and acid cannabinoids, respectively.

Cannabinoids standard solutions (CBDV, CBD, CBDA, CBC, CBG, CBGA, ( −)-Δ9-THC and THCA) were purchased from Merck (Darmstadt, Germany).

Orthophosphoric acid (85%) and HPLC-grade acetonitrile (ACN) were from Sigma-Aldrich (St. Louis, MI, USA).

A calibration curve was performed using cannabinoid standards with known concentrations, ranging from 0.5 to 100 μg/mL.

### Statistical analysis

VOC data were explored using the following statistical methods: volcano plot associated to *t*-test and fold change to compare the two-group data represented of freeze drying vs tray drying; analysis of variance (PerMANOVA and ANOVA) followed by Tukey’s test, principal component analysis (PCA) and random forest (RF) (MetaboAnalyst software version 5.0 and ChromCompare + software version 2.1.4) [53, 54] for the multi-group storage conditions. Two-dimensional hierarchical cluster analysis (HCA) was performed with the hclust function in R stat and visualised by MetaboAnalsyst v.5.0.

## Results and discussion

### Effect of drying conditions on metabolite profiles in cannabis inflorescences

**Fig. 1:**
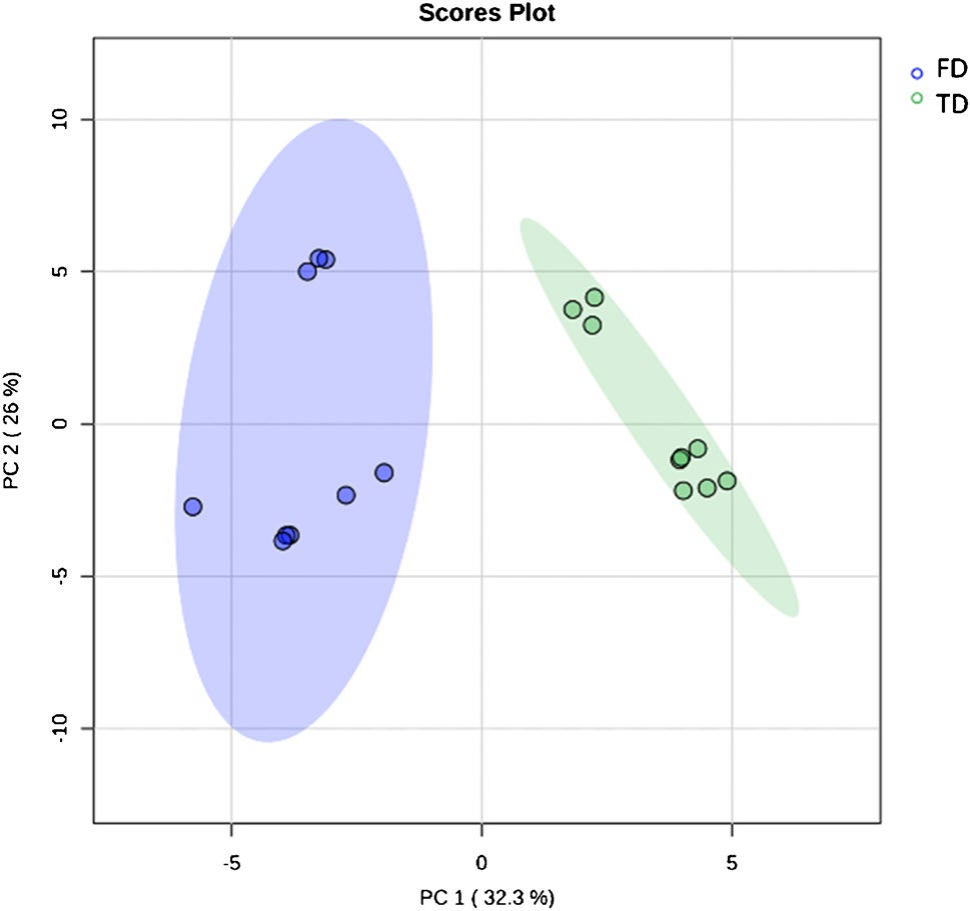
PCA score plot (PC1 and PC2) of overall metabolites profile (VOCs and cannabinoids) with 95% confidence interval, showing a variation between “tray drying” (TD, green area) and “freeze drying” (FD, light blue area)

The overall cannabinoid content and VOCs profile, measured for the three cannabis varieties, i.e. Kompolti (K), Eletta Campana (E), and Silvana (S) pooled and visualised by means of PCA score plot (PC1, representing 32.3% of variance, and PC2, 26%) with 95% confidence interval, highlighted a complete separation on PC1 between the samples subjected to “tray drying” (TD) compared to “freeze drying” (FD) conditions (Fig. 1), suggesting significant differences between the two drying methods. In this step, the differences were investigated as a whole, independently from the specific *C. sativa* variety to stress differences related to drying method on a wide range of samples instead of focusing on single variety variability.

**Fig. 2:**
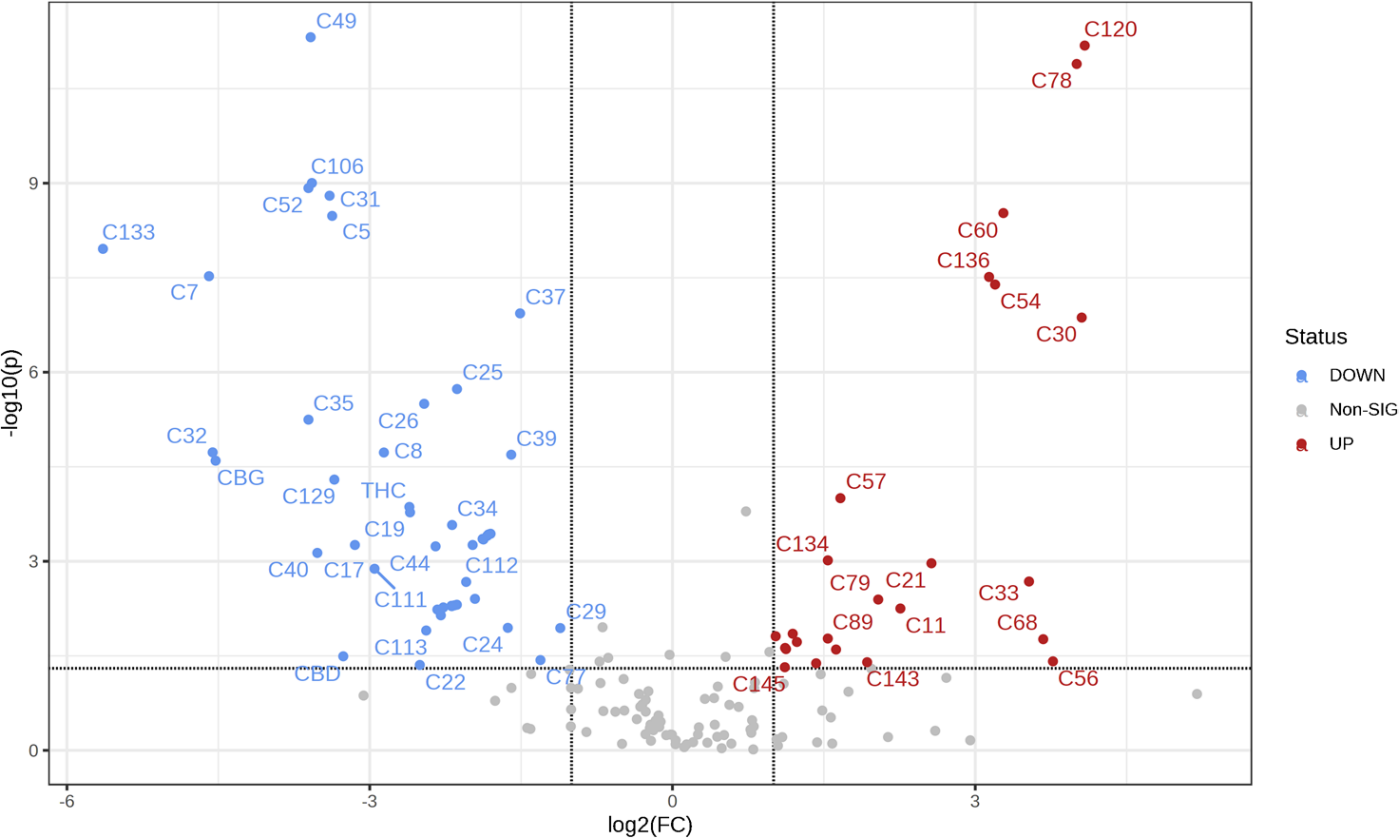
Volcano plot (log2fold change (FC) threshold > 2 on *x*-axis, and *t*-test *p*-value < 0.05 expressed in − log10 on *y*-axis) showing relevant differences between tray and freeze drying profiles. The blue compounds were detected at lower abundance in freeze-dried samples; the opposite was true for the red ones

To deeply investigate these changes between TD and FD, volcano plot analysis with a fold change threshold of 2 (log2, *x*-axis) and *T*-test *p*-value < 0.05 (− log10, *y*-axis) was performed with a total of 149 metabolites (Table S1). Among them, 69 metabolites showed significant differences between TD and FD conditions. As clearly shown in Fig. 2 and Table S2, the majority of metabolites (45) were detected at lower abundance in FD, while 24 metabolites showed the opposite trend with respect to TD processing.

**Fig. 3:**
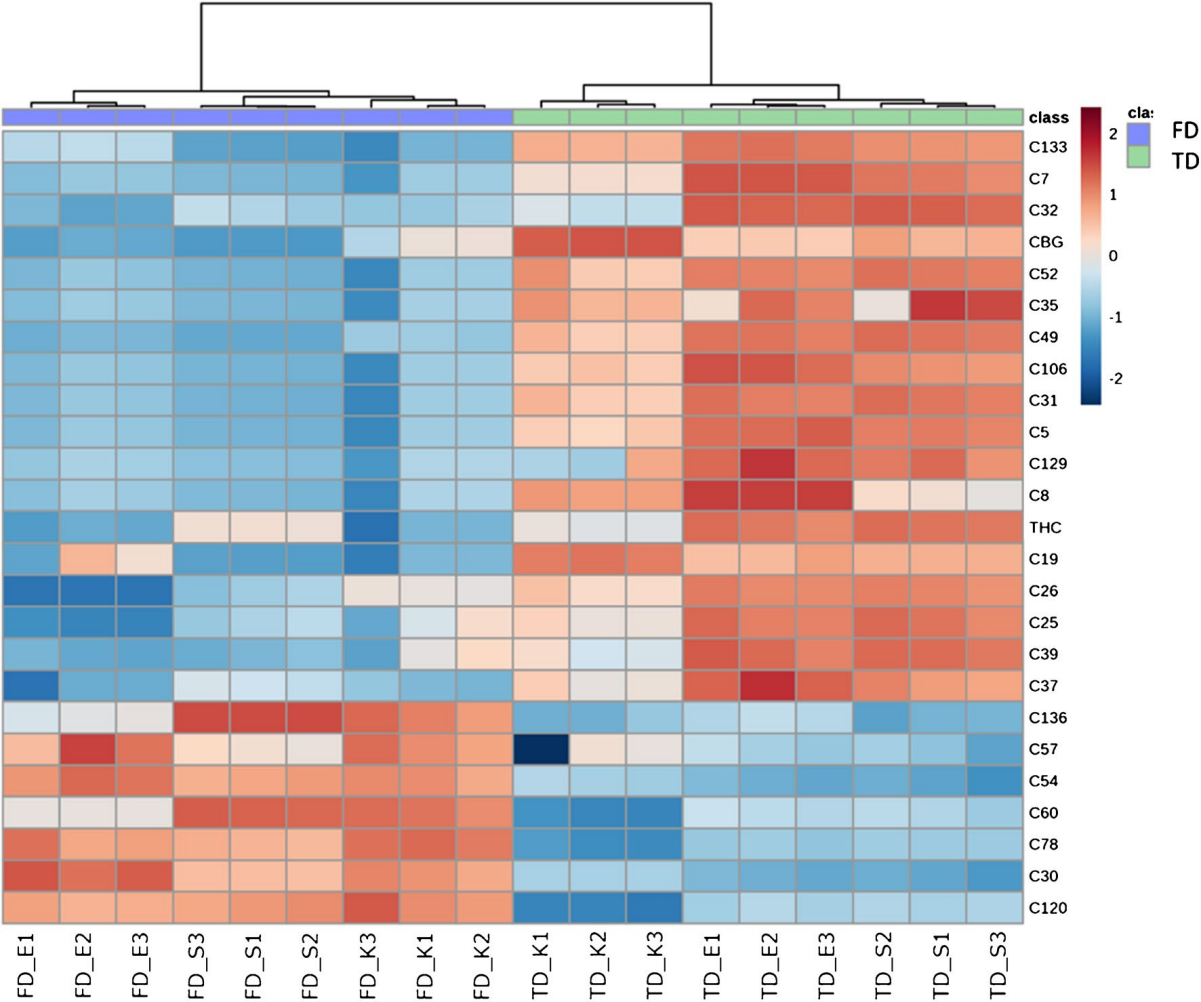
Hierarchical cluster analysis relative to cannabis inflorescences drying procedures FD and TD showing the top 25 most affected metabolites deriving from volcano plot analysis. Each coloured cell on the map corresponds to the relative measure of each metabolite (column normalised) after Log10 transformation to make metabolite intensity comparable. Cells in red show higher intensity, while those in blue refer to lower intensity. Sample codes refer to: freeze drying (FD), tray drying (TD); cannabis varieties Eletta Campana (E), Silvana (S), and Kompolti (K). The numbers 1, 2, and 3 refer to the biological sample

Among the 45 negatively affected compounds, 28 belonged to the terpene group, followed by six esters (two carboxylic acid and four fatty acid esters), three cannabinoids, three aldehydes, three alkanes, one furan, and one ketone. The 24 compounds that were positively affected by FD were of various groups: nine terpenes, four alkanes, three aldehydes, two esters and ketones, and one each of alcohols, aromatics, indoles, and one unknown. The top 25 most significant compounds are shown by means of heatmap in Fig. 3. Among them, the most negatively affected metabolites by FD were as follows: (-)-helminthogermacrene (C133), β-myrcene (C32), 3-carene (C35), butanal, 3-methyl- (C5), γ-bisabolene (C129), furan, 2-ethyl- (C8), 2-hexenal (C19), (E)-, camphene (C26), α-pinene (C25), d-limonene (C39), terpinolene (C37), and the two cannabinoids CBG and THC (Fig. 3 and Table S3). Few of the compounds found in the present work, namely, β-myrcene, α-pinene, d-limonene, and terpinolene, contribute to up of 50% of the aroma of cannabis [2, 55]. However, these compounds are relevant not only for their aromatic characteristics, but also because of other crucial pharmacological properties and the “entourage effect”, as described in introduction. Thus, it is clear that the FD technique results into a loss of the predominant cannabis key, multi-property, and aromatic compounds. The cold temperature used during freeze drying and ice crystal formation cause damage to the trichome microstructure [46]. This effect may increase the instant availability of aromatic VOCs resulting in the loss of these compounds during the time of the freeze drying process. Only seven compounds were among the top positively affected by FD (Table S3). Of these, only caryophyllene oxide (C136) and nonanal (C54) have been reported to impact the scent of cannabis [56].

Concerning cannabinoids, it is worth mentioning that the plant synthesises only the carboxylic acid form, namely, THCA, CBDA, and CBGA. Light and/or heat favour the formation of neutral forms through the decarboxylation of acidic cannabinoids, which are thermally unstable. The extent of the effects of the decarboxylation depends on the neutral to acidic ratio: the lower the ratio, the more relevant the % variation [57, 58]. In this case, despite a not significant change in acidic cannabinoids between TD and FD, the former processing type promotes the formation of neutral cannabinoids, i.e. CBD, CBG, and THC (Figs. 2 and 3), potentially due to the higher drying temperature. Therefore, conditions favouring decarboxylation are enhanced by TD.

In conclusion, FD can limit cannabinoid decarboxylation better than TD. However, it leads to a loss of predominant cannabis aroma compounds and of its distinctive scent. Furthermore, the loss of characterising terpenes may result in a bad appreciation by both the industry and the consumers. Based on these findings, FD does not represent an optimal alternative treatment to TD. For this reason, all the following sections report only results obtained after TD processing condition and upon storage treatments.

### *Cannabis sativa* VOC composition upon storage condition

The TD cannabis inflorescences were subjected to three storage conditions: (i) 500 mL V airtight box made of high-density polyethylene (HDPE), (ii) open to air dry tray (AT), and (iii) 250 mL lid tight brown glass bottle (GB). To reduce potential influence due to environmental parameters variability, all samples were stored in the same chamber in the dark, at controlled temperature (18 °C), humidity (60%) conditions, for 6 months. The range of VOCs produced by the TD samples before and after storage was accessed using a narrow varietal range, and then the presence of discriminating VOCs among the different storage treatments was evaluated. A total of 143 VOCs were putatively identified from all samples by retention index and spectral comparison with the NIST library; further, six unknown volatiles were also detected (Table S1). Compound groups included 81 terpenes (16 monoterpenes, 26 monoterpenoids, 27 sesquiterpenes, 12 sesquiterpenoids), 16 esters, 13 alcohols, eight alkanes, seven aldehydes, five ketones, four aromatics, three alkenes, three furans, one carboxylic acid, one indole, and one sulphur compound.

**Fig. 4:**
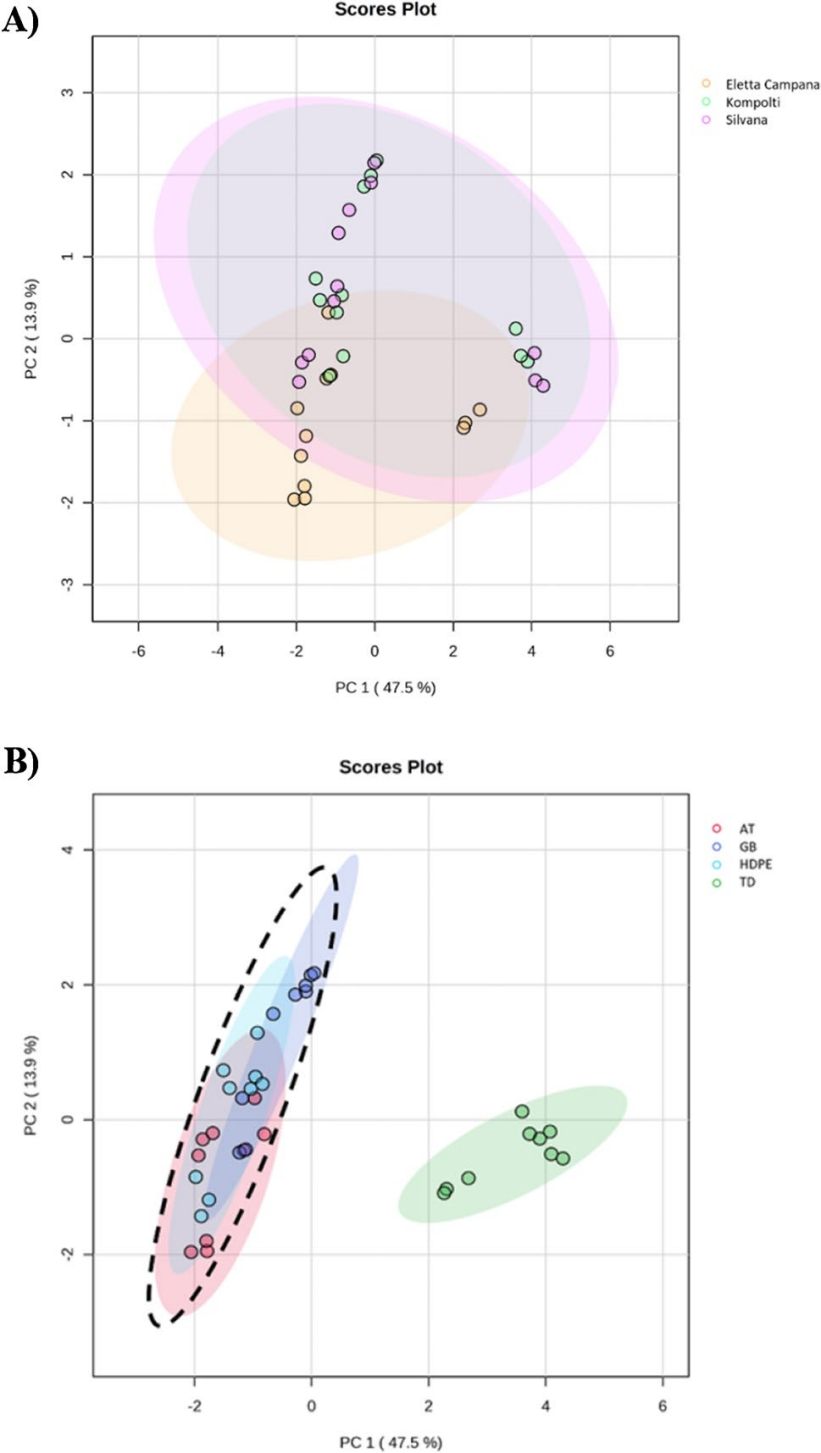
Principal component (PC) plots from PC analysis (PCA) based on all VOCs analysed using SPME-GC × GC–MS from three cannabis varieties: “Eletta Campana”, “Kompolti”, “Silvana” by **A** variety; **B** before storage (TD) and after storage (black dashed ellipse and GB, AT, HDPE ellipses). Each ellipse represents the 95% confidence interval. The plots use PC1 and PC2 with a percentage of explained variance of 61.4%

The pattern of VOC relative abundance changed greatly among samples and the variables accounted for the 78% of the variability based on PerMANOVA analysis. Significant differences were found before and after storage (PerMANOVA, *p* < 0.001, *R*^2^ = 0.34), amongst varieties (PerMANOVA, *p* < 0.001, *R*^2^ = 0.19), and for the interaction between varieties and storage (PerMANOVA, *p* < 0.001, *R*^2^ = 0.25). Furthermore, 50 variables resulted significantly different over the full set of samples (i.e. TD, GB, AT, HDPE; ANOVA, *p* < 0.05; Tukey’s post hoc test) (Table S4). Principal component (PC) plots produced from PCA analysis indicated that VOC profiles were not distinct among the Eletta Campana, Kompolti, and Silvana varieties when storage conditions were considered together (Fig. 4A).Conversely, a high degree of separation was obtained when VOC profiles of all varieties were plotted based on storage conditions. Indeed, inflorescences before storage (TD) can be clearly discriminated from after storage (GB, AT, HDPE), while after storage samples partially overlapped on the selected PCs (Fig. 4B).

**Fig. 5:**
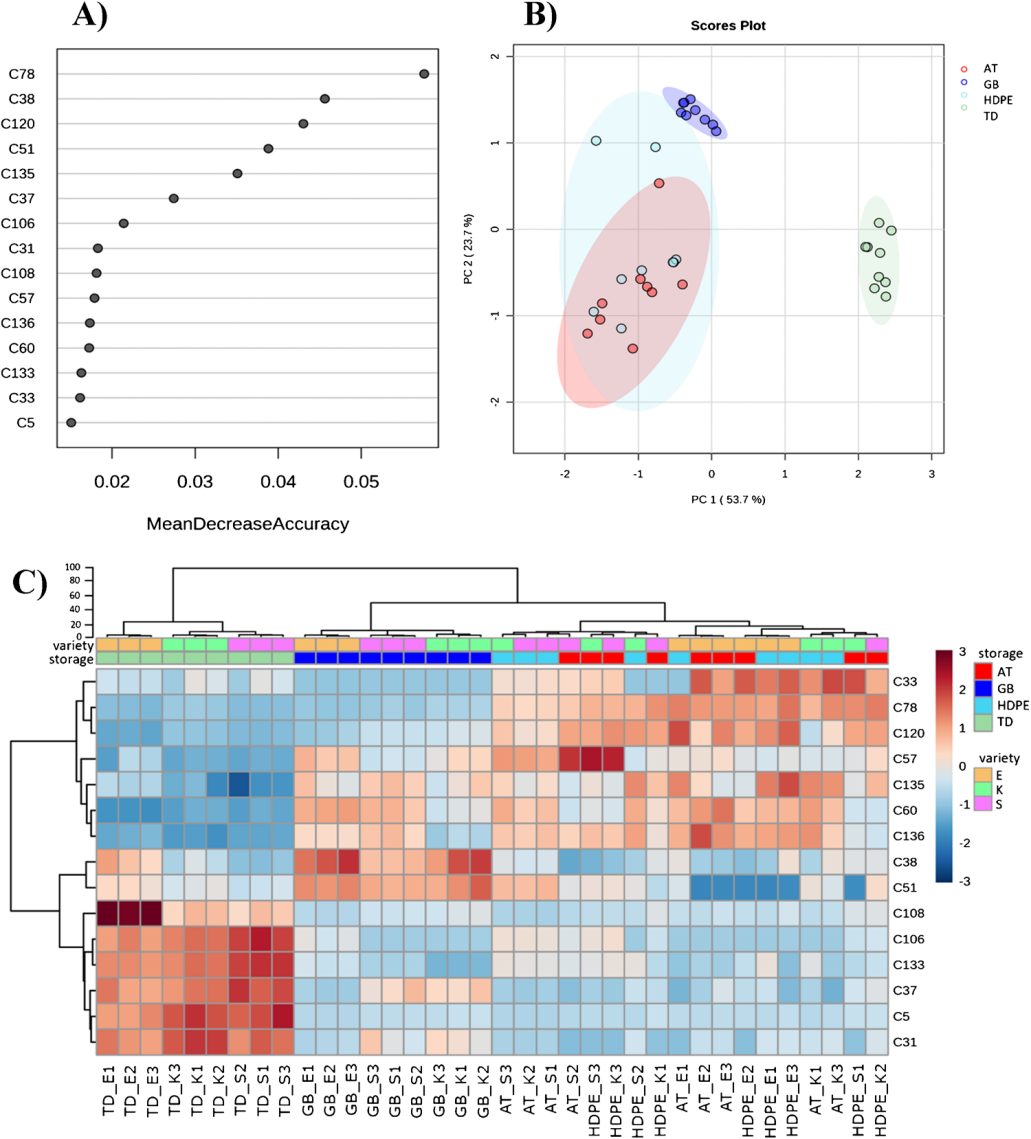
Results from random forest (RF) treatment classification based on VOCs. **A** Mean decrease accuracy analysis with VOCs ranched by their contribution to classification accuracy. **B** Principal component (PC) plots from PC analysis (PCA) based on RF VOCs of TD, GB, AT, and HDPE. Each ellipse represents the 95% confidence interval. The plots use PC1 and PC2 with a percentage of explained variance of 77.4%. **C** Heatmap and hierarchical clustering analysis obtained using the top discriminatory VOCs obtained with RF of the entire set of samples (see Table S1 compound names in italics). Sample codes refer to (i) tray drying (TD); (ii) airtight box made of high-density polyethylene (HDPE), (iii) open to air dry tray (AT), (iv) lid tight brown glass bottle (GB), (v) Eletta Campana (E), (vi) Kompolti (K), (vii) Silvana. The numbers 1, 2, and 3 refer to the biological sample. Blue to red colour on cells indicates low to high VOC abundance

Fifteen most discriminatory VOCs were identified using random forest (RF) for the differentiation across storage conditions (Fig. 5A, Table S5) comprising eight terpenes, two alkanes, one alcohol, one aldehyde, and one ketone. The overall out-of-bag (OOB) estimate of error rate was 0.055, while the class error rate was 0.11 for AT and HDPE and 0 for GB and TD. According to the confusion matrices derived from RF (Table 1), one AT sample was misclassified with a HDPE, and one of the HDPE was classified as AT. Furthermore, the principal component plot following RF showed a slight increase in explained variance for PC1 and PC2 (tot 77.4% compared to 61.4% for the complete data set). Moreover, the samples stored in GB were discriminated from those stored on AT; however, the latter overlapped with samples stored in HDPE (Fig. 5B). The 15 RF discriminatory VOCs were also analysed by hierarchical clustering using Euclidian distance and the Ward linkage clustering algorithm to minimise the sum of squares of any two clusters. The results visualised by means of heatmap and dendrogram confirmed the major influence on VOC profile changes due to storage; indeed as previously observed, the after storage preservation of the original VOCs profile can be very challenging [7]. Within storage conditions, samples stored in GB were still full separated from HDPE and AT ones, and the misclassification of HDPE and AT samples was confirmed (Fig. 5C, storage classification). Furthermore, the three varieties showed a minor impact on the changes in VOCs over the different treatments (Fig. 5C, variety classification). However, by looking at TD, the CBG-chemotype IV cannabis (E) showed a greater degree of separation with respect to CBD-chemotype III varieties (K, S), with p-cymene (C38), p-ocimene (C51), and perillyl acetate (C108), contributing to give a characteristic aroma to the CBG variety, Eletta Campana (E). Nevertheless, these differences became much less relevant when samples were subject to storage treatments.

**Table 1:** Confusion matrix derived from the random forest performed on the four conditions

	AT	GB	HDPE	TD	class.error
AT	8.00	0.00	1.00	0.00	0.11
GB	0.00	9.00	0.00	0.00	0.00
HDPE	1.00	0.00	8.00	0.00	0.11
TD	0.00	0.00	0.00	9.00	0.00

In this context, four main hierarchical clusters among volatile compounds were evident. The first cluster showed lower abundance (blue to white colour on cells) for most of the TD and GB samples, independently from the cannabis variety, and included the monoterpenoid (-)-myrtenol (C78), a compound of the cannabis volatile oils [59]. The second cluster included caryophyllene oxide (C136), a terpene and an essential oil having a significant impact on cannabis aroma [60], as well as nerolidol (C135), a cannabis volatile oil [59], and phenylethyl alcohol (C57) with a characteristic rose-like odour [61]. All these compounds showed lower abundance (blue colour) for TD samples [62], evidencing their formation after all storage treatments. The third cluster is accounted for p-cymene (C38) and p-ocimene (C51), compounds of the cannabis volatile oils [59] which showed higher abundance for GB samples if compared to TD and other storage methods. While the fourth cluster, which included terpinolene (C37), a cyclic monoterpene with a very distinctive smell [63], and (-)-helminthogermacrene (C133), found in a large variety of plants, including cannabis [64], showed higher abundance for most TD samples. This indicates that all storage methods lead to a reduction or degradation of all the compounds of the fourth cluster.

Boxplots of the relative abundance of each VOC within the treatments (i.e. TD, GB, AT, HDPE) are reported in supplementary Figure S1. All the compounds showed significant (*p* < 0.05) different profile between TD and the storage methods (GB, AT, and HDPE) with the exception of decane (C33), p-cymene (C38), p-ocimene (C51), and (-)-myrtenol (C78). In detail, decane (C33) and (-)-myrtenol (C78) were not significantly different between TD and GB, while p-cymene (C38) and p-ocimene (C51) did not differ among TD, AT, and HDPE. Seven compounds differed significantly between GB and AT, HDPE samples, an additional one differed between GB and HDPE, while only one between HDPE and AT. Among those p-cymene (C38), one of the main components of cumin essential oil, known for its storage stability [65], was significantly higher in GB. This may explain the different behaviour compared to other essential oils, like (-)-myrtenol (C78) and caryophyllene oxide (C136) high after HDPE and AT storage conditions. Indeed, both myrtenol and caryophyllene oxide derive from the oxidation of β-pinene and caryophyllene, reaction favoured by the presence of air [48].

TD treatment led to a prevalence of flavour and aroma compounds, such as perillyl acetate (C108), hexyl acetate (C37), methyl-butanal (C5), and pentamethylheptane (C31), and an intermediate compound in the biosynthesis of sesquiterpenes, namely germacrene (C133) [66]. Storage methods, on the other hand, lead to the enhancement of other flavour compounds, such as myrtenol (C78), phenylethyl alcohol (C57), and sesquiterpenoids, such as nerolidol (C135) and caryophyllene oxide (C136), having woody and sweet aroma and various biological properties including antimicrobial, antioxidant, anti-fungal, anticancer activities [66]. This indicates that storage methods contribute to change the initial aroma of cannabis inflorescences, due to the formation and/or degradation of defined analytes over time.

The similar VOC pattern for HDPE and AT may be linked to the permeability of the HDPE container. It has been demonstrated that plastic materials, conversely to glass, allow for water and oxygen permeability [67] from the external environment through the packaging and vice versa, thus leading to a similar environment between HDPE and AT storage conditions with an exchange of heat, moisture, and oxygen which in turn leads to oxidation reaction.

### Cannabinoid changes upon storage conditions

**Fig. 6:**
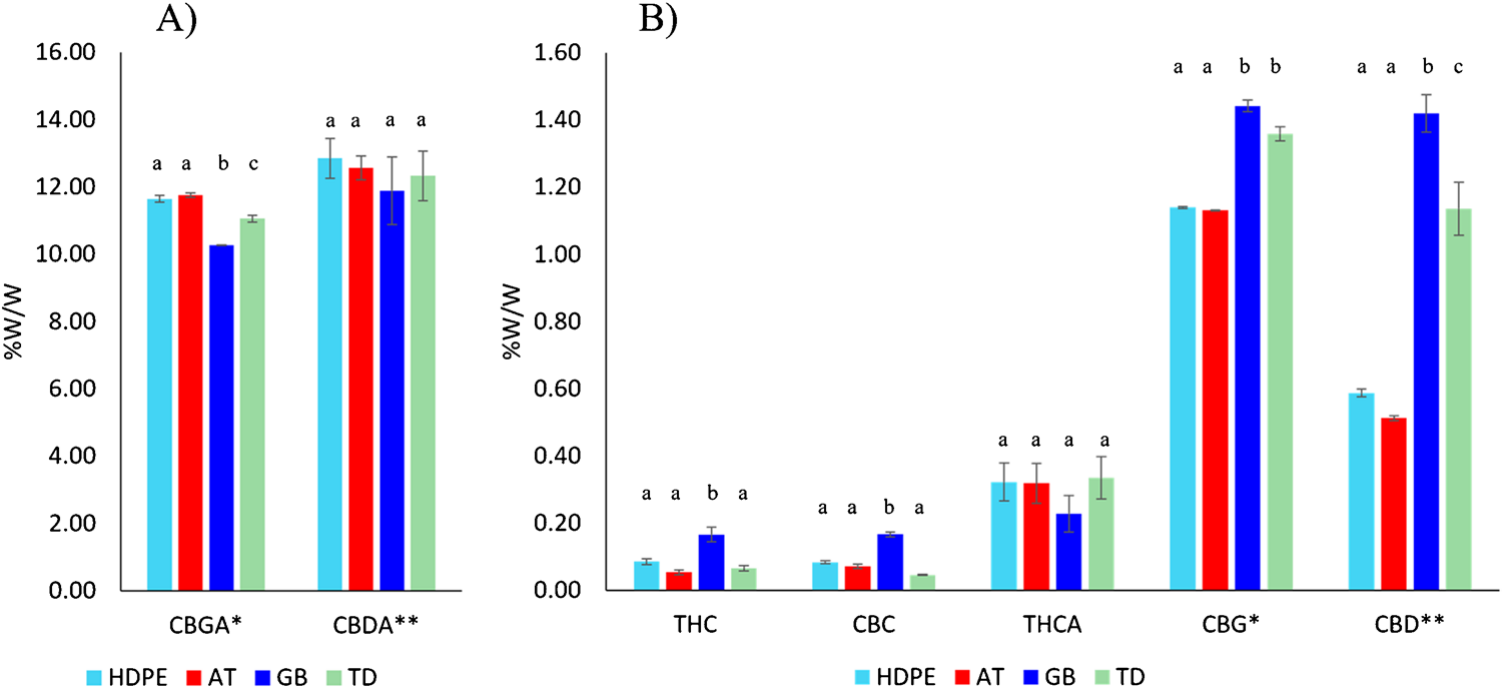
Barplot showing the normalised concentration of **A** THC, THCA, CBC, CBG, CBD; **B** CBGA and CBDA cannabinoids before (TD) and after storage in different conditions (HDPE, AT, and GB). * indicates CBG-chemotype IV; ** indicates CBD-chemotypes III. The same letter indicates that mean values are not significantly different from each other (*p* > 0.05)

Cannabinoid stability and deterioration is influenced by both biological and environmental factors including respiration, growth stage, water loss, pathological and physiological breakdown, light, temperature, relative humidity, and oxygen availability [40, 62, 68]. Moreover, it has been demonstrated that degradation and decarboxylation kinetics of cannabinoids depend on the nature of the target cannabinoid [58, 69]; hence, no generalisation can be made. As an example, THC degradation has a more chemical nature, while CBD degradation follows a more biochemical nature. In this context, the type and material of container used for the storage of cannabis plant products may play a pivotal role in the preservation of cannabinoids. In the following, the detailed investigation of the effect of storage conditions on cannabinoids content will be discussed. ANOVA statistical analysis was performed on the combined two varieties belonging to the CBD-chemotype III (Kompolti and Silvana) for the variables CBDA and CBD, while the third one, Eletta Campana, was treated separately, for the analysis of CBGA and CBG, since it is included in the CBG-chemotype IV. Therefore, the reported results for the above-mentioned cannabinoids derive from the average of the biological samples of two varieties or of the individual third one, respectively (Fig. 6 and Table S6). Minor cannabinoids (THC, THCA, and CBC) were collectively treated among all the cannabis varieties. Concerning the major acid cannabinoids, a similar trend is observed based on the storage method, where GB storage negatively influenced CBDA and CBGA content with respect to TD and the two other storage methods (Fig. 6A), albeit only the change in CBGA content was significantly different. As for the minor cannabinoid components, inflorescences stored under GB conditions showed a slight lower content in the acid cannabinoid THCA and a significant increase in content of the neutral cannabinoids (i.e. THC, CBC, CBG, and CBD) (Fig. 6B). For all cannabinoids, AT and HDPE performed equally, being able to maintain the initial cannabinoid concentrations found in TD for THC, CBC, THCA, and CBDA. In addition, the content of CBG and CBD was lower in AT and HDPE compared to TD and GB, while the opposite was evident for CBGA. As already discussed in the “Cannabis sativa VOC composition upon storage condition” section, glass, being the most inert material, represents a barrier against gases preventing any permeability. In this case, the closed environment inside the glass bottle cannot be in equilibrium with the external environment in terms of heat, moisture, and oxygen content; this may lead to an increase of decarboxylation event that in turn influence the increased formation of neutral cannabinoids (THC, CBC, CBD), as well as a decrease of the carboxylated forms (THCA and CBGA).

This result suggests a change of physiological conditions when inflorescences are stored in glass, highlighting the importance of conducting cannabinoids analysis when inflorescences of cannabis varieties are subjected to long storage. Furthermore, the selection of the material and storage condition will need further attention to better define an optimal storage method.

## Conclusions

A complete guideline concerning which type of postharvest cannabis treatments is best for preserving cannabinoids and cannabis aroma is still lacking. To shed light on this topic, in this study, a detailed investigation of the effects of different drying and storage preservation treatments has been presented for the first time through comprehensive VOC profiling and cannabinoids analysis of three cannabis varieties using SPME-GC × GC–MS and HPLC approaches, respectively. It has been demonstrated that tray drying (TD) is the best method to reduce water content in inflorescences while maintaining a rich VOC profile. Conversely, freeze drying method, being useful to preserve cannabinoid content, preventing decarboxylation of acid cannabinoids, leads to a loss of volatile compounds responsible for the characteristic aroma of cannabis. From the results, the scent of cannabis changes with time independently of the storage condition. Indeed, the volatilome of cannabis inflorescences after drying (TD) is not maintained upon any of the storage conditions applied, i.e. open to air container, HDPE box, and glass bottle. Storage in HDPE and in open to air containers (AT) leads to very a similar volatile organic compound pattern, evidencing that oxidation reactions of defined molecules are favoured in these conditions if compared to glass. However, within storage, minor differences can be seen with the formation of new compounds based on the individual storage condition. Some of these compounds are particularly relevant, being characterised by sensorial notes and belonging to the essential oil of the plant. Many of these belong to the terpenes, sesquiterpenoid, and terpenoids subgroups (e.g. nerolidol and terpinolene) and may contribute to give a characteristic inflorescence scent depending on the selected storage condition. Concerning cannabinoid content, storage in a glass bottle leads to a more marked increase in neutral cannabinoids and a decrease of acid cannabinoids if compared to the other two storage methods. Overall, storage in glass may be more beneficial for the minor change of the initial VOC content if compared to the other storage conditions. However, the same conclusion cannot be applied for the cannabinoid content, suggesting that an optimal preservation condition for the retention of both VOCs and cannabinoid is still not available.

Therefore, depending on the scope of inflorescence preservation, one may select the most convenient storage method. Further studies are extremely important for the definition of optimal storage conditions of cannabis inflorescences, useful for the production of highly valuable and high-quality products, with significant implications on the market.

### Supplementary Information

Below is the link to the electronic supplementary material.

Supplementary file1 (PDF 576 KB)

## Data Availability

The data and material generated during and/or analysed during the current study are available from the corresponding author upon reasonable request.
