# Supplementary material for: The influence of drying and storage conditions on the volatilome and cannabinoid content of Cannabis sativa L. inflorescences
Source: Anal Bioanal Chem. 2024 May 3;416(16):3797–809. doi: 10.1007/s00216-024-05321-w (PMC11180634; doi:10.1007/s00216-024-05321-w)
Supplement: Supplementary file 1 — Supplementary file1 (PDF 576 KB) [file 216_2024_5321_MOESM1_ESM.pdf]

The influence of drying and storage conditions on the volatilome and cannabinoid content of *Cannabis sativa* L inflorescences

SUPPLEMENTARY MATERIAL

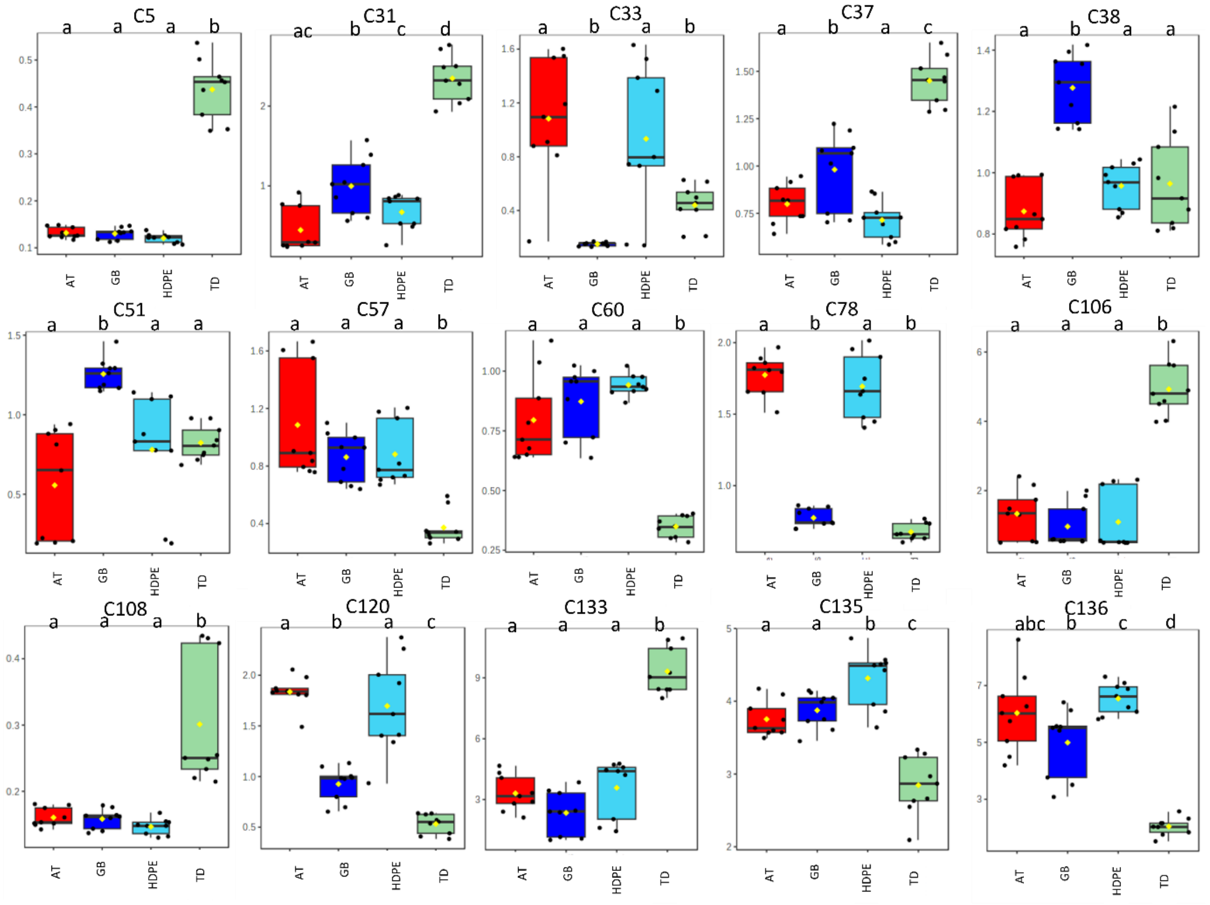

Figure S1: Boxplots of the selected VOCs based on Random Forest analysis in tray drying (TD, light green) condition, and after 6 months storage in airtight box made of high-density polyethylene (HDPE,light blue), lid tight brown glass bottle (GB, dark blue), open to air dry tray (AT,red). The same letter indicates that mean values are not significantly different from each other ($p > 0.05$). Different letters indicate significant differences ($p<0.05$, see Tables S1 and S5 for details). The black dots represent the relative intensity of the selected compounds from all samples. The mean relative intensity of each group is indicated with a yellow diamond.

Table S1: Complete list of VOCs detected in all cannabis samples.

Code	VOC	RI	CAS	Compound family	Compound Sub group
C1	Acetone	487	67-64-1	ketones	
C2	Dimethyl sulfide	520	75-18-3	sulfur compound	
C3	Acetic acid	610	64-19-7	carboxylic acids	
C4	1,3-Pentadiene, 2-methyl-	624	1118-58-7	alkene	
C5	*Butanal, 3-methyl-*	*652*	*590-86-3*	*aldehydes*	
C6	1-Propene, 3-propoxy-	684	1471-03-0	alkenes	
C7	Butanal, 2-methyl-	662	96-17-3	aldehydes	
C8	Furan, 2-ethyl-	703	3208-16-0	furans	
C9	Propanoic acid, ethyl ester	712	105-37-3	esters	carboxylic acid esters
C10	Propanoic acid, 2-methyl-, ethyl ester	756	97-62-1	esters	fatty acid esters
C11	Hexane, 2,3-dimethyl-	760	584-94-1	alkanes	
C12	Methyl isovalerate	776	556-24-1	esters	fatty acid esters
C13	Octane	800	111-65-9	alkanes	
C14	Hexanal	801	66-25-1	aldehydes	
C15	Butanoic acid, ethyl ester	802	105-54-4	esters	fatty acid esters
C16	2-Butenoic acid, 3-methyl-, methyl ester	842	924-50-5	esters	fatty acids esters
C17	Butanoic acid, 2-methyl-, ethyl ester	849	7452-79-1	esters	fatty acid esters
C18	Butanoic acid, 3-methyl-, ethyl ester	853	108-64-5	esters	fatty acid esters
C19	2-Hexenal, (E)-	854	6728-26-3	aldehydes	
C20	1-Hexanol	868	111-27-3	alcohols	
C21	Heptanal	901	111-71-7	aldehydes	

C22	5,5-Dimethyl-1-vinylbicyclo[2.1.1]hexane	921	16626-39-4	alkanes	
C23	2-Butenoic acid, 3-methyl-, ethyl ester	924	638-10-8	esters	fatty acid esters
C24	α-Thujene	929	2867-05-02	terpenes	monoterpenes
C25	α-Pinene	937	80-56-8	terpenes	monoterpenes
C26	Camphene	952	79-92-5	terpenes	monoterpenes
C27	2,4(10)-Thujadiene	956	36262-09-6	terpenes	monoterpenes
C28	Benzaldehyde	962	100-52-7	aldehydes	
C29	β-Pinene	943	18172-67-3	terpenes	monoterpenes
C30	Sulcatone	986	110-93-0	ketones	
C31	*2,2,4,6,6-Pentamethylheptane*	*990*	*13475-82-6*	*alkanes*	
C32	β-Myrcene	991	123-35-3	terpenes	monoterpenes
C33	*Decane*	*1000*	*124-18-5*	*alkanes*	
C34	α-Phellandrene	1005	99-83-2	terpenes	monoterpenes
C35	3-Carene	1011	13466-78-9	terpenes	monoterpenes
C36	Acetic acid, hexyl ester	1011	142-92-7	esters	carboxylic esters
C37	*Terpinolene*	*1088*		*terpenes*	*monoterpenes*
C38	*p-Cymene*	*1025*	*99-87-6*	*terpenes*	*monoterpenes*
C39	D-Limonene	1031	5989-27-5	terpenes	monoterpenes
C40	β-Phellandrene	1031	555-10-2	terpenes	monoterpenes
C41	Eucalyptol	1032	470-82-6	terpenes	monoterpenoids
C42	Benzyl alcohol	1036	100-51-6	alcohols	
C43	β-Ocimene	1049	3779-61-1	terpenes	monoterpenes
C44	Unknown 2	1049			
C45	γ-Terpinene	1060	99-85-4	terpenes	monoterpenes
C46	4-Thujanol	1070	17699-16-0	terpenes	monoterpenoids
C47	1-Octanol	1070	111-87-5	alcohols	fatty alcohols
C48	Linalool oxide	1074	5989-33-3	furans	
C49	α-Terpinolene	1088	586-62-9	terpenes	monoterpenes

C50	Fenchone	1096	1195-79-5	terpenes	monoterpenoids
C51	*p-Ocimene*	*1090*	*1195-32-0*	*terpenes*	*monoterpenes*
C52	Undecane	1100	1120-21-4	alkanes	
C53	Linalool	1099	78-70-6	terpenes	monoterpenoids
C54	Nonanal	1104	124-19-6	aldehydes	
C55	Propanoic acid, hexyl ester	1108	2445-76-3	esters	fatty acid esters
C56	Acetic acid, heptyl ester	1112	112-06-1	esters	fatty acid esters
C57	*Phenylethyl Alcohol*	*1116*	*60-12-8*	*alcohols*	
C58	Fenchol	1114	1632-73-1	terpenes	monoterpenoids
C59	trans-2-Pinanol	1132	4948-29-2	terpenes	monoterpenoids
C60	*4-Acetyl-1-methylcyclohexene*	*1137*	*6090-09-1*	*ketones*	
C61	L-Pinocarveol	1139	547-61-5	alcohols	
C62	Benzyl nitrile	1144	140-29-4	aromatics	
C63	2-Pinanol	1132	4948-29-2	terpenes	monoterpenoids
C64	Ipsdienol	1147	35628-00-3	alcohols	
C65	Camphor	1145	76-22-2	terpenes	monoterpenoids
C66	Myrcenone	1145	539-70-8	ketones	
C67	Camphene hydrate	1148	465-31-6	terpenes	monoterpenoids
C68	1,5,7-Octatrien-3-ol, 2,6-dimethyl-	1167	29414-56-0	terpenes	monoterpenoids
C69	2-Methyl-6-methylene-1,7-octadien-3-one	1145	22459-10-5	terpenes	monoterpenoids
C70	endo-Borneol	1167	507-70-0	terpenes	monoterpenoids
C71	Pinocamphone	1160	547-60-4	terpenes	monoterpenoids
C72	Terpinen-4-ol	1182	20126-76-5	terpenes	monoterpenoids
C73	p-Cymen-8-ol	1183	1197-01-9	alcohols	phenylpropanes
C74	4-Methylacetophenone	1183	122-00-9	ketones	
C75	n-Hexyl butanoate	1192	2639-63-6	esters	fatty acid esters
C76	α±-Terpineol	1189	98-55-5	terpenes	monoterpenoids

C77	Dodecane	1200	112-40-3	alkanes	
C78	*(-)-Myrtenol*	*1213*	*19894-97-4*	*terpenes*	*monoterpenoids*
C79	Acetic acid, octyl ester	1210	112-14-1	esters	carboxylic acid esters
C80	Fenchyl acetate	1224	13851-11-1	terpenes	monoterpenoids
C81	Carveol	1219	99-48-9	terpenes	monoterpenoids
C82	α-Citronellol	1213	6812-78-8	alcohols	
C83	Hexyl 2-methylbutyrate	1236	10032-15-2	esters	fatty acid esters
C84	(-)-Carvone	1245	6485-40-1	terpenes	monoterpenoids
C85	5-Decen-1-ol, (E)-	1247	56578-18-8	alcohols	
C86	(Z)-4-Decen-1-ol	1257	57074-37-0	alcohols	
C87	Myrtanol	1261	51152-12-6	terpenes	monoterpenoids
C88	Bornyl acetate	1285	76-49-3	terpenes	monoterpenoids
C89	Indole	1295	120-72-9	Indoles	
C90	Benzene, (2-nitroethyl)-	1304	6125-24-2	aromatics	
C91	Myrtenyl acetate	1327	1079-01-2	terpenes	monoterpenoids
C92	2,4-Decadien-1-ol	1307	14507-02-9	alcohols	
C93	Tridecane	1300	629-50-5	alkanes	
C94	Methyl anthranilate	1343	134-20-3	esters	carboxylic acid ester
C95	Citronellyl acetate	1353	150-84-5	terpenes	monoterpenoids
C96	8-Hydroxylinalool	1361	64142-78-5	alcohols	
C97	Copaene	1376	3856-25-5	terpenes	sesquiterpenes
C98	Ylangene	1372	14912-44-8	terpenes	sesquiterpenes
C99	Myrtanyl acatate	1387	29021-36-1	terpenes	monoterpenoids
C100	Hexyl caproate	1384	6378-65-0	esters	fatty acid esters
C101	Alloisolongifolene	1409	87064-18-4	terpenes	sesquiterpenes
C102	Selina-5,11-diene	1447	52026-55-8	terpenes	sesquiterpenes
C103	α-Gurjunene	1409	489-40-7	terpenes	sesquiterpenes
C104	Caryophyllene	1419	87-44-5	terpenes	sesquiterpenes

C105	α-Bergamotene	1435	17699-05-7	terpenes	sesquiterpenes
C106	*Unknown 3*	*1454*			
C107	Germacrene D	1481	23986-74-5	terpenes	sesquiterpenes
C108	*Perillyl acetate*	*1436*	*15111-96-3*	*terpenes*	*monoterpenoids*
C109	8,10-Dodecadien-1-ol	1473	33956-49-9	alcohols	
C110	Aromandendrene	1440	489-39-4	terpenes	sesquiterpenes
C111	Bicyclo[3.1.1]heptane, 6-methyl-2-methylene-6-(4-methyl-3-pentenyl)-, [1R-(1α,5α,6β)]-	1446	55123-21-2	terpenes	monoterpenoids
C112	γ-Gurjunene	1473	22567-17-5	terpenes	sesquiterpenes
C113	(-)-Aristolene	1447	6831-16-9	terpenes	sesquiterpenes
C114	Humulene	1454	6753-98-6	terpenes	sesquiterpenes
C115	α-Farnesene	1508	502-61-4	terpenes	sesquiterpenes
C116	Alloaromadendrene	1461	25246-27-9	terpenes	sesquiterpenes
C117	α-Elemene	1454	5951-67-7	terpenes	sesquiterpenes
C118	4,5-Di-epi-aristolochene	1470	54868-40-5	terpenes	sesquiterpenes
C119	β-Guaiene	1490	88-84-6	terpenes	sesquiterpenes
C120	*Unknown*	*1500*			
C121	Naphthalene, 1,2,3,4,4a,5,6,7-octahydro-4a,8-dimethyl-2-(1-methylethenyl)-	1492	103827-22-1	aromatics	
C122	Valencene	1492	4630-07-3	terpenes	sesquiterpenes
C123	α±-Farnesene	1508	502-61-4	terpenes	sesquiterpenes
C124	Dihydroagarofuran	1550	5956-12-7	furans	
C125	β-Curcumene	1514	28976-67-2	terpenes	sesquiterpenes

C126	α-Selinene	1494	473-13-2	terpenes	sesquiterpenes
C127	β-Bisabolene	1509	495-61-4	terpenes	sesquiterpenes
C128	β-Sesquiphellandrene	1524	20307-83-9	terpenes	sesquiterpenes
C129	γ-Bisabolene	1514	13062-00-5	terpenes	sesquiterpenes
C130	Naphthalene, decahydro-4a-methyl-1-methylene-7-(1-methylethylidene)-	1544	58893-88-2	aromatics	
C131	α-Bisabolene	1512	25532-79-0	terpenes	sesquiterpenes
C132	Selina-3,7(11)-diene	1542	6813-21-4	terpenes	sesquiterpenes
C133	*(-)-helminthogermacrene*	*1570*	*75023-40-4*	*terpenes*	*sesquiterpenes*
C134	Unknown 1	1577			
C135	*Nerolidol*	*1563*	*7212-44-4*	*terpenes*	*sesquiterpenoids*
C136	*Caryophyllene oxide*	*1581*	*1139-30-6*	*terpenes*	*sesquiterpenoids*
C137	Guaiol	1596	489-86-1	terpenes	sesquiterpenoids
C138	α-epi-7-epi-5-Eudesmol	1616	446050-56-2	terpenes	sesquiterpenoids
C139	Humulene oxide II	1606	19888-34-7	terpenes	sesquiterpenoids
C140	Unknown 4	1662			
C141	Agarospirol	1644	19431-80-2	terpenes	sesquiterpenoids
C142	Hinesol	1635	23811-08-7	terpenes	sesquiterpenoids
C143	β-Selinenol	1649	473-15-4	terpenes	sesquiterpenoids
C144	Unknown 5	1653			
C145	7-epi-a-Eudesmol	1658	123123-38-6	terpenes	sesquiterpenoids
C146	8-Heptadecene	1675	2579-04-6	alkenes	
C147	Bulnesol	1667	22451-73-6	terpenes	sesquiterpenoids
C148	Levomenol	1695	23089-26-1	terpenes	sesquiterpenoids
C149	Eudesm-7(11)-en-4-ol	1692	473-04-1	terpenes	sesquiterpenoids

In italics the top discriminatory VOCs obtained with RF.

Table S2: Significant metabolites based on Volcano plot analysis. FC=fold change, raw.pval= p-value based on t-test, FD=freeze drying.

Feature Name	Chemical Group	Chemical subgroup	Code	FC	log2(FC)	raw.pval	LOG10(p)
**Compounds negatively affected by FD**							
2-Hexenal, (E)-	aldehydes		C19	0.16	-2.60	0.00	3.78
Butanal, 3-methyl-	aldehydes		C5	0.10	-3.37	0.00	8.48
Butanal, 2-methyl-	aldehydes		C7	0.04	-4.59	0.00	7.52
5,5-Dimethyl-1-vinylbicyclo[2.1.1]hexane	alkanes		C22	0.18	-2.50	0.04	1.36
2,2,4,6,6-Pentamethylheptane	alkanes		C31	0.09	-3.40	0.00	8.80
Undecane	alkanes		C52	0.08	-3.61	0.00	8.92
Dodecane	alkanes		C77	0.40	-1.31	0.04	1.43
CBD	Cannabinoids		CBD	0.10	-3.26	0.03	1.49
CBG	Cannabinoids		CBG	0.04	-4.53	0.00	4.60
THC	Cannabinoids		THC	0.16	-2.61	0.00	3.86
Propanoic acid, 2-methyl-, ethyl ester	esters	carboxylic acid esters	C10	0.23	-2.14	0.00	2.31
Propanoic acid, ethyl ester	esters	carboxylic acid esters	C9	0.22	-2.19	0.01	2.29
Butanoic acid, ethyl ester	esters	fatty acid esters	C15	0.22	-2.16	0.00	2.30
Butanoic acid, 2-methyl-, ethyl ester	esters	fatty acid esters	C17	0.11	-3.15	0.00	3.26
Propanoic acid, hexyl ester	esters	fatty acid esters	C55	0.20	-2.30	0.01	2.14
Methyl isovalerate	esters	fatty acid esters	C12	0.22	-2.19	0.01	2.29
Furan, 2-ethyl-	furans		C8	0.14	-2.86	0.00	4.73
Myrcenone	ketones		C66	0.20	-2.33	0.01	2.23
α-Phellandrene	terpenes	monoterpenes	C34	0.22	-2.18	0.00	3.58
3-Carene	terpenes	monoterpenes	C35	0.08	-3.61	0.00	5.25
Terpinolene	terpenes	monoterpenes	C37	0.35	-1.51	0.00	6.93
D-Limonene	terpenes	monoterpenes	C39	0.33	-1.60	0.00	4.69
β-Phellandrene	terpenes	monoterpenes	C40	0.09	-3.52	0.00	3.13
α-Terpinolene	terpenes	monoterpenes	C49	0.08	-3.59	0.00	11.32
α-Thujene	terpenes	monoterpenes	C24	0.32	-1.63	0.01	1.94
α-Pinene	terpenes	monoterpenes	C25	0.23	-2.13	0.00	5.73
Camphene	terpenes	monoterpenes	C26	0.18	-2.46	0.00	5.50
2,4(10)-Thujadiene	terpenes	monoterpenes	C27	0.28	-1.84	0.00	3.40
β-Pinene	terpenes	monoterpenes	C29	0.46	-1.11	0.01	1.94
β-Myrcene	terpenes	monoterpenes	C32	0.04	-4.56	0.00	4.73
Perillyl acetate	terpenes	monoterpenoids	C108	0.29	-1.80	0.00	3.44
Camphor	terpenes	monoterpenoids	C65	0.28	-1.82	0.00	3.43
Myrtenyl acetate	terpenes	monoterpenoids	C91	0.27	-1.87	0.00	3.35
Bicyclo[3.1.1]heptane, 6-methyl-2-methylene-6-(4-methyl-3-pentenyl)-, [1R-(1α,5α,6β)]-	terpenes	monoterpenoids	C111	0.13	-2.95	0.00	2.88

2-Methyl-6-methylene-1,7-octadien-3-one	terpenes	monoterpenoids	C69	0.21	-2.27	0.01	2.27
Fenchyl acetate	terpenes	monoterpenoids	C80	0.27	-1.88	0.00	3.36
Alloisolongifolene	terpenes	sesquiterpenes	C101	0.25	-1.98	0.00	3.26
γ-Gurjunene	terpenes	sesquiterpenes	C112	0.24	-2.04	0.00	2.67
(-)-Aristolene	terpenes	sesquiterpenes	C113	0.18	-2.44	0.01	1.90
α-Elemene	terpenes	sesquiterpenes	C117	0.26	-1.96	0.00	2.40
β-Bisabolene	terpenes	sesquiterpenes	C127	0.22	-2.19	0.01	2.29
γ-Bisabolene	terpenes	sesquiterpenes	C129	0.10	-3.35	0.00	4.30
(-)-helminthogermacrene	terpenes	sesquiterpenes	C133	0.02	-5.64	0.00	7.96
Unknown 3			C106	0.08	-3.57	0.00	9.00
Unknown 2			C44	0.20	-2.35	0.00	3.24
**Compounds positively affected by FD**							
Phenylethyl Alcohol	alcohols		C57	3.17	1.66	0.00	4.00
Heptanal	aldehydes		C21	5.92	2.57	0.00	2.97
Benzaldehyde	aldehydes		C28	2.35	1.23	0.02	1.72
Nonanal	aldehydes		C54	9.17	3.20	0.00	7.39
Hexane, 2,3-dimethyl-	alkanes		C11	4.78	2.26	0.01	2.25
Unknown			C120	16.96	4.08	0.00	11.18
Decane	alkanes		C33	11.58	3.53	0.00	2.68
Benzyl nitrile	aromatics		C62	2.03	1.02	0.02	1.81
Acetic acid, heptyl ester	esters	fatty acid esters	C56	13.64	3.77	0.04	1.41
Acetic acid, octyl ester	esters	fatty alcohols esters	C79	4.11	2.04	0.00	2.39
Indole	Indoles		C89	2.91	1.54	0.02	1.77
Sulcatone	ketones		C30	16.62	4.05	0.00	6.87
4-Acetyl-1-methylcyclohexene	ketones		C60	9.71	3.28	0.00	8.53
1,5,7-Octatrien-3-ol, 2,6-dimethyl-	terpenes	monoterpenoids	C68	12.76	3.67	0.02	1.76
α±-Terpineol	terpenes	monoterpenoids	C76	2.17	1.12	0.02	1.63
(-)-Myrtenol	terpenes	monoterpenoids	C78	16.06	4.01	0.00	10.89
Caryophyllene oxide	terpenes	sesquiterpenoids	C136	8.80	3.14	0.00	7.51
Guaiol	terpenes	sesquiterpenoids	C137	2.18	1.12	0.02	1.61
α-epi-7-epi-5-Eudesmol	terpenes	sesquiterpenoids	C138	2.68	1.42	0.04	1.38
β-Selinenol	terpenes	sesquiterpenoids	C143	3.81	1.93	0.04	1.40
7-epi-a-Eudesmol	terpenes	sesquiterpenoids	C145	2.16	1.11	0.05	1.32
Unknown 1			C134	2.91	1.54	0.00	3.01
Unknown 4			C140	2.28	1.19	0.01	1.85
Unknown 5			C144	3.08	1.62	0.03	1.60

Supplementary table S3. Top 25 most significant metabolites (blu= negatively affected by freeze drying, red=positively affected by freeze drying) with their aroma description. FC=fold change, raw.pval= p-value based on t-test

Feature Name	Chemical Group	Chemical subgroup	Code	FC	log2(FC)	raw.pval	LOG10(p)	*Aroma descriptors
α-Terpinolene	terpenes	monoterpenes	C49	0.083328	-3.5851	4.82E-12	11.317	woody, woody earthy spicy
Unknown 3			C106	0.084053	-3.5726	9.95E-10	9.0023	
Undecane	alkanes		C52	0.082067	-3.6071	1.20E-09	8.9225	musty, green
2,2,4,6,6-Pentamethylheptane	alkanes	branched alkanes	C31	0.094904	-3.3974	1.58E-09	8.801	cedar and musk fragrance
Butanal, 3-methyl-	aldehydes	aldehydes	C5	0.096598	-3.3719	3.30E-09	8.4811	gasoline-like to odorless
(-)-Helminthogermacrene	terpenes	sesquiterpenes	C133	0.02001	-5.6432	1.10E-08	7.9585	woody
Butanal, 2-methyl-	aldehydes	aldehydes	C7	0.041443	-4.5927	2.99E-08	7.5246	pine-like
Terpinolene	terpenes	monoterpenes	C37	0.35112	-1.51	1.16E-07	6.934	caramel, buttery, baked bread, and nutty
α-Pinene	terpenes	monoterpenes	C25	0.22768	-2.1349	1.85E-06	5.7329	fruity
Camphene	terpenes	monoterpenes	C26	0.18167	-2.4606	3.17E-06	5.499	malty
3-Carene	terpenes	monoterpenes	C35	0.082083	-3.6068	5.65E-06	5.2478	balsamic, sweet, myrrh, orange flower
β-Myrcene	terpenes	monoterpenes	C32	0.042494	-4.5566	1.87E-05	4.7287	ethereal rum, cocoa note
Furan, 2-ethyl-	furans	furans	C8	0.13776	-2.8598	1.88E-05	4.7268	earthy, woody and herbaceous odor
D-Limonene	terpenes	monoterpenes	C39	0.33039	-1.5978	2.03E-05	4.692	green
CBG	Cannabinoids	Cannabinoids	CBG	0.043373	-4.5271	2.53E-05	4.5971	camphor-like
γ-Bisabolene	terpenes	sesquiterpenes	C129	0.098041	-3.3505	5.05E-05	4.2967	dry woody, resinous-piney
THC	Cannabinoids	Cannabinoids	THC	0.16412	-2.6072	0.0001372	3.8626	citrus-like
2-Hexenal, (E)-	aldehydes	aldehydes	C19	0.16484	-2.6009	0.0001671	3.777	pine-like
Unknown			C120	16.963	4.0843	6.54E-12	11.184	sweet, fresh, dry wood, spicy
(-)-Myrtenol	terpenes	monoterpenes	C78	16.055	4.005	1.28E-11	10.892	sweet and fruity

4-Acetyl-1-methylcyclohexene	ketones	ketones	C60	9.7075	3.2791	2.98E-09	8.5259	spice
Caryophyllene oxide	terpenes	sesquiterpenes	C136	8.7982	3.1372	3.07E-08	7.5126	herbal, minty
Nonanal	aldehydes	aldehydes	C54	9.1685	3.1967	4.05E-08	7.3921	citrus-like lemongrass
Sulcatone	ketones	ketones	C30	16.617	4.0546	1.36E-07	6.8675	
Phenylethyl Alcohol	alcohols	aromatic alcohols	C57	3.1691	1.6641	9.96E-05	4.0019	floral, sweet, with rose note

*Aroma descriptors were sourced from Good Scents company database (https://www.thegoodscentscompany.com/search3.php.1988)

Table S4: Significant variables based on ANOVA and Tukey's post-hoc test. TD=tray drying, HDPE=airtight box made of high-density polyethylene, AT=air dry tray, GB=lid tight brown glass bottle; p.value <0.05

Code	f.value	p.value	FDR	Tukey's HSD				
C5	294.47	2.2581e-23	3.2968e-21	TD-AT	GB-TD	HDPE-TD		
C78	237.56	6.1146e-22	4.4636e-20	TD-AT	GB-AT	GB-TD	HDPE-TD	HDPE-GB
C60	77.40	9.3275e-15	4.5394e-13	TD-AT	GB-TD	HDPE-TD		
C120	69.26	4.3694e-14	1.5948e-12	TD-AT	GB-AT	GB-TD	HDPE-TD	HDPE-GB
C136	65.83	8.7963e-14	2.5685e-12	TD-AT	GB-TD	HDPE-TD	HDPE-GB	
C8	46.51	9.0304e-12	2.1974e-10	TD-AT	GB-TD	HDPE-TD		
C37	37.36	1.4404e-10	3.0042e-09	TD-AT	GB-TD	HDPE-TD	HDPE-GB	
C7	34.87	3.3483e-10	6.1107e-09	TD-AT	GB-TD	HDPE-TD		
C31	32.28	8.4684e-10	1.3738e-08	TD-AT	GB-AT	GB-TD	HDPE-TD	
C108	30.72	1.526e-09	2.228e-08	TD-AT	GB-TD	HDPE-TD		
C52	29.46	2.4903e-09	3.1346e-08	TD-AT	GB-AT	GB-TD	HDPE-TD	HDPE-GB
C135	29.37	2.5764e-09	3.1346e-08	TD-AT	HDPE-AT	GB-TD	HDPE-TD	
C57	27.46	5.5842e-09	6.2715e-08	TD-AT	GB-TD	HDPE-TD		
C133	23.28	3.4966e-08	3.6464e-07	TD-AT	GB-TD	HDPE-TD		
C32	21.95	6.5932e-08	6.4173e-07	TD-AT	GB-TD	HDPE-TD		
C38	20.77	1.1746e-07	1.0613e-06	GB-AT	GB-TD	HDPE-GB		
C94	20.67	1.2358e-07	1.0613e-06	TD-AT	GB-TD	HDPE-TD		
C106	19.69	2.0368e-07	1.6521e-06	TD-AT	GB-TD	HDPE-TD		
C35	15.31	2.3645e-06	1.783e-05	TD-AT	GB-TD	HDPE-TD		
C49	15.26	2.4425e-06	1.783e-05	TD-AT	GB-TD	HDPE-TD		
C33	15.00	2.8576e-06	1.9867e-05	TD-AT	GB-AT	GB-TD	HDPE-GB	
C92	14.38	4.1802e-06	2.7741e-05	TD-AT	GB-TD	HDPE-TD		
C15	14.19	4.7296e-06	3.0023e-05	TD-AT	GB-TD	HDPE-TD		
C12	14.06	5.119e-06	3.0906e-05	TD-AT	GB-TD	HDPE-TD		
C9	14.01	5.2921e-06	3.0906e-05	TD-AT	GB-TD	HDPE-TD		
C69	13.66	6.5932e-06	3.7023e-05	TD-AT	GB-TD	HDPE-TD		
C40	13.59	6.9348e-06	3.7499e-05	TD-AT	GB-TD	HDPE-TD		
C4	10.90	4.3369e-05	0.0002261	HDPE-AT	GB-TD	HDPE-TD		
C105	10.37	6.368e-05	0.0003206	TD-AT	GB-TD	HDPE-TD		

C122	10.04	8.1343e-05	0.0003959	TD-AT	GB-TD	HDPE-TD	
C26	9.51	0.0001222	0.0005755	TD-AT	GB-TD	HDPE-TD	
C93	8.12	0.0003681	0.0016795	TD-AT	GB-AT		
C70	7.69	0.0005254	0.0023245	TD-AT	GB-TD	HDPE-TD	
C89	7.29	0.0007348	0.0031552	GB-AT	HDPE-AT	GB-TD	HDPE-TD
C59	6.94	0.0009946	0.0041487	TD-AT	GB-TD	HDPE-TD	
C25	6.43	0.0015653	0.006348	TD-AT	HDPE-TD		
C51	6.30	0.001753	0.0068931	GB-AT			
C58	6.28	0.0017941	0.0068931	TD-AT	GB-TD	HDPE-TD	
C28	6.05	0.002212	0.0082807	TD-AT			
C111	5.91	0.0025	0.0091251	HDPE-TD			
C74	5.38	0.0040971	0.01459	TD-AT	GB-TD		
C14	5.32	0.0043405	0.015088	GB-TD			
C30	5.26	0.0045839	0.015564	TD-AT			
C45	5.04	0.0056648	0.018797	TD-AT	HDPE-TD		
C62	4.81	0.0071021	0.023043	GB-AT	GB-TD		
C124	4.65	0.0083112	0.026379	TD-AT	HDPE-TD		
C27	4.52	0.0094624	0.029394	TD-AT			
C140	4.48	0.0098234	0.02988	TD-AT	HDPE-TD		
C24	4.37	0.010923	0.032547	TD-AT			
C34	4.18	0.013202	0.038549	TD-AT			

Table S5: RF 15 VOC relative abundance based on different storage conditions

	VOC %Rel. abundance	C5	C31	C33	C37	C38	C51	C57	C60	C78	C106	C108	C120	C133	C135	C136
HDPE	average	0.12	0.67	0.93	0.71	0.96	0.78	0.88	0.94	1.69	1.09	0.15	1.70	3.58	4.32	6.54
	SE	0.00	0.07	0.19	0.03	0.02	0.12	0.07	0.02	0.08	0.29	0.00	0.16	0.48	0.13	0.18
AT	average	0.13	0.45	1.08	0.80	0.87	0.56	1.09	0.79	1.77	1.33	0.16	1.84	3.31	3.75	6.03
	SE	0.00	0.09	0.15	0.03	0.03	0.11	0.13	0.06	0.05	0.24	0.00	0.05	0.29	0.08	0.46
GB	average	0.13	1.00	0.15	0.98	1.28	1.26	0.86	0.87	0.77	0.96	0.16	0.93	2.34	3.87	4.99
	SE	0.00	0.12	0.00	0.07	0.04	0.03	0.06	0.05	0.02	0.21	0.01	0.06	0.36	0.08	0.40
TD	average	0.44	2.35	0.44	1.45	0.96	0.82	0.37	0.35	0.68	4.93	0.30	0.53	9.32	2.85	2.04
	SE	0.02	0.10	0.05	0.04	0.05	0.04	0.04	0.02	0.02	0.26	0.03	0.03	0.38	0.14	0.10

Table S6: Cannabinoids content based on different storage conditions.

	% w/w	CBGA*	CBDA**	THC	CBC	THCA	CBG*	CBD**
HDPE	average	11.65	12.86	0.07	0.07	0.27	1.14	0.59
	SE	0.10	0.59	0.01	0	0.04	0	0.01
AT	average	11.76	12.57	0.05	0.07	0.32	1.13	0.51
	SE	0.07	0.36	0	0	0.04	0	0.01
GB	average	10.27	11.89	0.17	0.17	0.24	1.44	1.42
	SE	0.01	1.00	0.02	0	0.04	0.02	0.06
TD	average	11.65	12.34	0.07	0.05	0.36	1.43	1.14
	SE	0.11	0.74	0.01	0	0.05	0.02	0.08
